# Distribution of self-reported borderline personality disorder traits symptoms in a large-scale clinical population

**DOI:** 10.3389/fpsyt.2024.1424966

**Published:** 2024-06-25

**Authors:** Yong Lin, ZiLei Guo, Yong Zhou, YanYan Wei, LiHua Xu, XiaoChen Tang, Zixuan Wang, YeGang Hu, JiJun Wang, Yi Mei, HaiSu Wu, YanLi Luo, TianHong Zhang

**Affiliations:** ^1^ Department of Psychiatry, Kangci Hospital of Jiaxing, Tongxiang, Zhejiang, China; ^2^ Shanghai Mental Health Center, Shanghai Jiaotong University School of Medicine, Shanghai Engineering Research Center of Intelligent Psychological Evaluation and Intervention, Shanghai Key Laboratory of Psychotic Disorders, Shanghai, China; ^3^ Department of Psychology, Shanghai Xinlianxin Psychological Counseling Center, Shanghai, China; ^4^ Department of Psychological Medicine, Renji Hospital, School of Medicine, Shanghai Jiao Tong University, Shanghai, China

**Keywords:** personality disorder, borderline trait, self-report, mood disorders, schizophrenia

## Abstract

**Introduction:**

Borderline Personality Disorder (BPD) traits play a crucial role in the prognosis of psychiatric disorders, as well as in assessing risks associated with negativity and impulsivity. However, there is a lack of data regarding the distribution characteristics of BPD traits and symptoms within clinical populations.

**Methods:**

A total of 3015 participants (1321 males, 1694 females) were consecutively sampled from outpatients at the psychiatric and psycho-counseling clinics at the Shanghai Mental Health Center. BPD symptoms were assessed using a self-reported personality diagnostic questionnaire. Having BPD traits is defined as having five or more positive items in self-reported BPD characteristics. Participants were stratified into male and female groups, age groups, and diagnostic groups (schizophrenia, mood disorders, anxiety disorders). Exploratory factor analysis using principal components analysis was conducted. Three factors were identified: “F1: Affective Instability and Impulsivity”, “F2: Interpersonal Unstable and Extreme Reactions”, and “F3: Identity Disturbance”.

**Results:**

Among 3015 participants, 45.9% of the patients self-reported BPD traits. Comparing of male and female patients, there was no statistically significant difference in the occurrence rate of BPD traits (*χ^2^ =* 1.835, *p*=0.176). However, in terms of symptoms, female patients reported more symptoms than male patients. Female patients also exhibited more pronounced features on F2 compared to male patients (*t* =-1.972, *p*=0.049). There is a general decrease in BPD traits, symptoms, and factors with increasing age. Specifically, the proportion of positive BPD traits is approximately halved before the age of 30 and decreases to around one-third after the age of 30. BPD traits were most common in the Mood Disorders group at 55.7%, followed by the Anxiety Disorders group at 44.4%, and Schizophrenia group at 41.5% (*χ^2^ =* 38.084, *p*<0.001).

**Discussion:**

Our study revealed the pervasive presence of BPD traits and symptoms among psychiatric outpatients, exhibiting distinctive distributions across gender, age, and diagnostic categories. These findings emphasize the significance of identifying and addressing BPD pathology in the clinical care of psychiatric outpatients.

## Introduction

Borderline Personality Disorder (BPD) is a common personality disorder characterized by pervasive emotion dysregulation, impulsivity, identity disturbances, and unstable and intense interpersonal relationships (1–3). Individuals with BPD are at an elevated risk of early death over the course of the disorder (4). Longitudinal research has reported rates of completed suicide and rates of early death from other medical causes in the longer term. A study by Temes et al. (5) followed 290 adult BPD inpatients over a 24-year period and found that 5.9% of borderline patients died by suicide, indicating an increased risk of premature death among individuals with BPD. Despite the significant risk associated with BPD, it remains a challenging disorder both in terms of diagnosis and treatment (6). There is ongoing controversy regarding its conceptualization, with debate over whether it should be viewed as a specific personality disorder or as a level of impairment in general personality functioning (7). Treatment for BPD, including both pharmacotherapy (8) and psychotherapy (9), remains challenging.

In the identification of BPD, instability in multiple aspects such as self-image, emotions, interpersonal relationships, and behavioral patterns is involved. These unstable features often blur the boundaries between BPD and other psychiatric disorders, leading to frequent comorbidities (10–12). These comorbidities contribute to poor prognosis. A previous longitudinal study tracking a clinical cohort with BPD found that chronic somatic conditions are prevalent in individuals with BPD and interact negatively with persistent BPD pathology, deteriorating their long-term quality of life (13). In addition to comorbidities with Axis I disorders and physical illnesses, comorbidities between BPD and other personality disorders are also common. It suggests that BPD exhibits features of personality dysfunction that are shared across all types of personality disorders and exist on a severity dimension (14).

Previous research has demonstrated that BPD traits and symptoms manifest differently across genders and ages (15, 16). Studies have shown mixed results regarding gender differences in BPD (17, 18). Some studies indicate that certain BPD features, such as suicidal behavior and intense anger, are more prevalent in women, while other studies report no significant gender differences. For instance, Johnson et al. found that women with BPD tend to exhibit more emotional dysregulation and self-harm behaviors than men (19), whereas Zanarini et al. found no significant gender differences in the overall severity of BPD symptoms (20). Age-related changes in BPD symptoms have also been observed. Longitudinal studies suggest that certain BPD symptoms, such as impulsivity and aggression, tend to decrease with age. For example, research by Paris and Zweig-Frank indicated that older individuals with BPD exhibit fewer impulsive behaviors and have a lower prevalence of substance use disorders compared to younger individuals (3).

In recent years, the prevalence of BPD patients and BPD traits has been increasing among psychiatric clinical populations, particularly among adolescents and young adults (21), with Non-Suicidal Self-Injury as a prominent feature (22). This situation poses significant challenges to clinical diagnosis and treatment. Overall, BPD traits are characterized by impulsivity and the risk of negative self-harm and suicide. Additionally, they often coexist with other psychiatric disorders, exerting a significant impact on the prognosis of common mental disorders. Therefore, it is crucial for clinicians to recognize and address BPD traits as an important component of routine assessments. Given these concerns, this study investigated the distribution of BPD traits among a large clinical sample, stratifying by gender, age, and diagnosis, aiming to achieve a better understanding of BPD’s presentation in psychiatric clinical populations.

## Methods

### Sample and procedures

The survey was conducted at the Shanghai Mental Health Center (SMHC) from 2016 to 2023, involving participants recruited from outpatients attending psycho-counseling and psychiatric clinics at SMHC, one of China’s largest healthcare facilities. The study received approval from the Research Ethics Committee at SMHC (2016–3R), and participants provided written informed consent during the recruitment stage. The study aimed to assess the prevalence of Personality Disorders (PDs) in a consecutive clinical sample of adult patients. A total of 3015 outpatients were randomly selected between January 2016 and December 2023, meeting inclusion criteria such as age between 18 and 55 years, ability to comprehend the study questionnaire, willingness to disclose information about PDs, and being under stable treatment conditions. “Stable treatment conditions” are defined as having no changes in treatment regimen, including medication and therapy, in the two weeks prior to study enrollment. Exclusion criteria included severe or unstable physical conditions, current pregnancy, and other factors identified by investigators as rendering the patient ineligible.

Among the screened participants, 219 individuals did not complete the self-assessment scales, resulting in incomplete data and their exclusion from the analysis. Primary reasons for non-completion included perceived assessment duration being too lengthy for 179 participants, 33 individuals providing entirely inconsistent responses, 3 participants refusing researchers’ use of information provided after the interview, and 7 individuals requesting to withdraw from the study without providing a clear reason. Ultimately, data from 3015 patients were included in the final analysis. Among 3015 participants, the mean age was 31.5 years (SD=9.679), with 1694 females (56.2%), 1592 being first diagnosed (52.8%), 1551 single (51.4%), and the mean duration of disorder being 58.9 months (SD=75.798).

### BPD trait measurements

The assessment of BPD traits and symptoms utilized a concise and well-structured self-report questionnaire, namely the Personality Diagnostic Questionnaire 4th Edition Plus (PDQ-4plus) (23), as described in previous publications (2, 10, 24). The PDQ-4plus comprises 107 true-false questions designed to evaluate 10 Axis II DSM-IV PDs, including BPD, which is the focus of this study. Specifically, the questionnaire includes 11 items related to BPD traits, corresponding to the 9 diagnostic criteria outlined in DSM-IV (see **Table 1**). Having BPD traits is defined as having five or more positive items in self-reported BPD characteristics, corresponding to the requirement in BPD diagnostic criteria of meeting five or more diagnostic criteria. The primary aim of the PDQ-4plus is to differentiate individuals exhibiting characteristics associated with PD from those who do not. It demonstrates high sensitivity (0.89) and acceptable specificity (0.65). Widely used for screening DSM-IV PDs in Chinese psychiatric patients, the PDQ-4plus has shown high test-retest reliability (0.92) within the Chinese population, indicating the questionnaire’s reliability in yielding consistent results (11, 25, 26).

**Table 1:** Correspondence of BPD Diagnostic Criteria to PDQ-4plus-C Items and Three Factors.

Diagnostic criteria	PDQ-4plus-C items	Factors
**1. **Frantic efforts to avoid real or imagined abandonment	To prevent the people I love from leaving me, I would go to extremes. (Item-6) Once I realize that someone close to me is no longer getting close to me, I feel very upset and make various strong reactions. (Item-100)	F2: Interpersonal Unstable and Extreme Reactions
**2. **A pattern of unstable and intense interpersonal relationships	I either like or admire someone, or I resent them, without any feelings in between. (Item-19) My relationships with others sometimes become very intimate, and sometimes become full of resentment. (Item-101)	F2: Interpersonal Unstable and Extreme Reactions
**3. **Identity disturbance	I often want to figure out who I really am. (Item-32)	F3: Identity Disturbance
**4. **Impulsivity in at least two areas that are potentially self-damaging (e.g., spending, sex, substance abuse, reckless driving, binge eating)	I have done some of the following things on impulse and gotten myself into trouble. a. Spent more money than I could afford; b. Had sex with someone I didn’t know well; c. Drank too much alcohol; d. Used drugs; e. Overeaten; f. Reckless biking or driving. (Item-106)	F1: Affective Instability and Impulsivity
**5. **Recurrent suicidal behavior, gestures, or threats, or self-mutilating behavior	I have tried to hurt myself or commit suicide. (Item-45)	F2: Interpersonal Unstable and Extreme Reactions
**6. **Affective instability due to a marked reactivity of mood	I am a person with unstable emotions. (Item-58)	F1: Affective Instability and Impulsivity
**7. **Chronic feelings of emptiness	I feel that my life is dull and meaningless. (Item-69)	F3: Identity Disturbance
**8. **Inappropriate, intense anger or difficulty controlling anger (e.g., frequent displays of temper, constant anger, recurrent physical fights)	I have difficulty controlling my anger or temper. (Item-78)	F1: Affective Instability and Impulsivity
**9. **Transient, stress-related paranoid ideation or severe dissociative symptoms	When faced with stressful situations, I become sensitive, suspicious, or forgetful about things I just did. (Item-93)	F3: Identity Disturbance

### Clinical diagnosis

All participants received clinical diagnoses from their psychiatrists according to routine clinical practice, utilizing the International Classification of Diseases, Tenth Edition (ICD-10). The diagnoses were provided by psychiatrists with over 5 years of clinical experience, ensuring a high level of diagnostic reliability and expertise. Participants diagnosed with schizophrenia were classified under the F20 category, which includes various subtypes and categories such as paranoid schizophrenia (F20.0), hebephrenic schizophrenia (F20.1), catatonic schizophrenia (F20.2), undifferentiated schizophrenia (F20.3), residual schizophrenia (F20.5), other schizophrenia (F20.8), and unspecified schizophrenia (F20.9).

Participants diagnosed with mood disorders were categorized under the F30-F39 range, encompassing various subtypes and categories including manic episode (F30), bipolar affective disorder (F31), depressive episode (F32), recurrent depressive disorder (F33), persistent mood disorders (F34), other mood disorders (F38), and unspecified mood disorders (F39).

Participants diagnosed with anxiety disorders were classified under the F40-F48 range, which includes various subtypes and categories such as phobic anxiety disorders (F40), other anxiety disorders (F41), obsessive-compulsive disorder (F42), reaction to severe stress, and adjustment disorders (F43), dissociative disorders (F44), somatoform disorders (F45), and other nonpsychotic mental disorders (F48).

### Data analysis

Data analysis was performed using SPSS version 20.0 (SPSS, Inc., Chicago, IL, USA). The dataset underwent double data entry, and rigorous data checking and cleaning procedures were conducted to ensure both range and consistency prior to analysis. Statistical significance was set at p < 0.05. Quantitative variables are presented as mean ± standard deviation (SD), while qualitative variables are presented as frequencies (%). We conducted exploratory factor analysis using principal components analysis, followed by varimax rotation with Kaiser normalization. The determination of the number of factors retained in the analysis was based on factors with eigenvalues greater than 1. Subsequently, utilizing the factor loading coefficients, we calculated the estimated factor scores for each factor for all participants. Three factors were identified: “F1: Affective Instability and Impulsivity”, “F2: Interpersonal Unstable and Extreme Reactions”, and “F3: Identity Disturbance”. Participants were stratified into male and female groups, age groups (18–20, 21–25, 26–30, 31–35, 36–40, 41–45, 46–50, 51–55 years), and diagnostic groups (Schizophrenia, mood disorders, anxiety disorders). Differences between means and proportions were assessed using the t-test (for comparison between two groups), one-way ANOVA (for comparison among three or more groups), and the Chi-square test, respectively. To visually depict gender and age differences more intuitively, bar charts and line graphs were utilized.

## Results

### Criteria and factors

**Figure 1:**
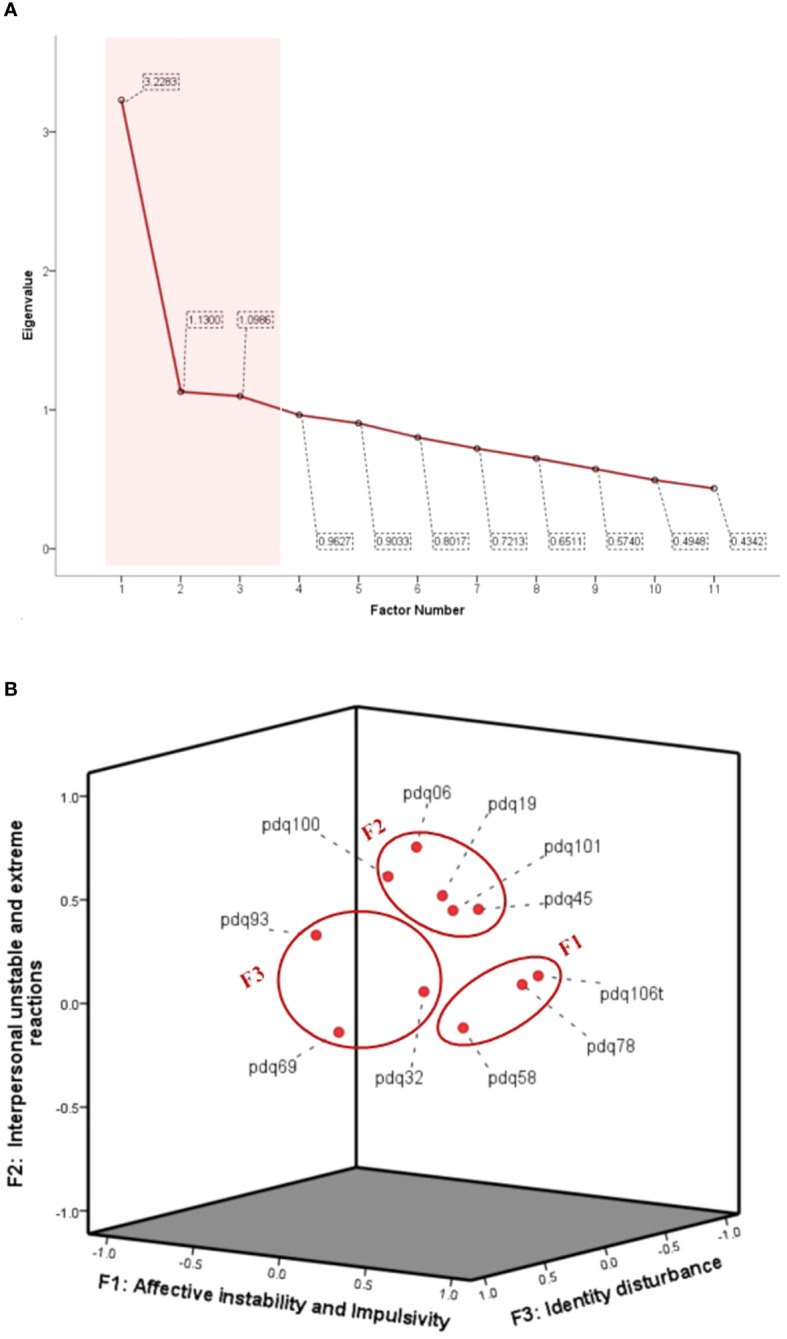
Factors by self-reported BPD traits. **(A)** Standardized factor loadings were obtained from the exploratory factor analysis, using varimax rotation. **(B)** Factor plot in rotated space.

**Table 1** presents the BPD diagnostic criteria alongside their corresponding PDQ-4plus-C items. Exploratory factor analysis of the PDQ-4plus-C items revealed three factors, as illustrated in **Figures 1A, B**. These three factors exhibited eigenvalues greater than 1, indicating their significance, while other factors with eigenvalues below 1 were not retained (**Figure 1A**). The first factor, with an eigenvalue of 3.228, comprised items 58, 78, and 106, and was labeled as “Affective Instability and Impulsivity”. The second factor, with an eigenvalue of 1.130, included items 6, 19, 45, 100, and 101, and was termed “Interpersonal Unstable and Extreme Reactions”. The third factor, with an eigenvalue of 1.099, encompassed items 32, 69, and 93, and was designated as “Identity Disturbance” (**Figure 1B**).

### Profiles of BPD criteria and factors in overall sample and gender groups

As shown in **Table 2**, nearly half (45.9%) of the clinically assessed patients self-reported BPD traits. Among our sample population, the most commonly reported BPD symptom was Criterion 6 “Affective instability due to a marked reactivity of mood” (66.9%), followed by Criterion 9 “Transient, stress-related paranoid ideation or severe dissociative symptoms” (63.5%). Additionally, the more severe symptom “Recurrent suicidal behavior, gestures, or threats, or self-mutilating behavior” was reported by almost one-third of the patients (31.6%). Comparing BPD traits and symptoms reported by male and female patients, there was no statistically significant difference in the occurrence rate of BPD traits between the two groups (*χ^2^* = 1.835, *p* = 0.176). However, in terms of symptoms, female patients reported more Criteria 1 (*χ^2^* = 6.208, *p* = 0.013), 5 (*χ^2^* = 14.691, *p* < 0.001), and 8 (*χ^2^* = 12.655, *p* < 0.001) than male patients. Female patients also exhibited more pronounced features on Factor 2 “Interpersonal Unstable and Extreme Reactions” compared to male patients (*t* = -1.972, *p* = 0.049).

**Table 2:** Frequency of BPD trait, criteria, and profile of three factors, compared between genders.

	Overall (N=3015)	Female (N=1694)	Male (N=1321)	*χ^2^/t*	*P*
BPD trait (Criteria >=5) [n (%)]	1384 (45.9%)	796 (47.0%)	588 (44.5%)	***χ^2^*** = 1.835	0.176
Diagnostic criteria [n (%)]					
Criteria 1	1586 (52.6%)	925 (54.6%)	661 (50.0%)	*χ^2^* = 6.208	**0.013**
Criteria 2	1400 (46.4%)	772 (45.6%)	628 (47.5%)	*χ^2^* = 1.155	0.283
Criteria 3	1410 (46.8%)	774 (45.7%)	636 (48.1%)	*χ^2^* = 1.796	0.180
Criteria 4	1169 (38.8%)	642 (37.9%)	527 (39.9%)	*χ^2^* = 1.245	0.265
Criteria 5	953 (31.6%)	584 (34.5%)	369 (27.9%)	*χ^2^* = 14.691	**<0.001**
Criteria 6	2018 (66.9%)	1154 (68.1%)	864 (65.4%)	*χ^2^* = 2.477	0.116
Criteria 7	1335 (44.3%)	745 (44.0%)	590 (44.7%)	*χ^2^* = 0.141	0.707
Criteria 8	1553 (51.5%)	921 (54.4%)	632 (47.8%)	*χ^2^* = 12.655	**<0.001**
Criteria 9	1915 (63.5%)	1092 (64.5%)	823 (62.3%)	*χ^2^* = 1.496	0.221
Factors [Mean (SD.)]					
Factor 1	–	0.020 (1.000)	-0.025 (1.003)	*t* = -1.2221	0.222
Factor 2	–	0.031 (1.027)	-0.040 (0.963)	*t* = -1.972	**0.049**
Factor 3	–	0.005 (0.979)	-0.006 (1.027)	*t* = -0.298	0.766

Diagnostic Criteria: 1. Frantic efforts to avoid real or imagined abandonment; 2. A pattern of unstable and intense interpersonal relationships; 3. Identity disturbance; 4. Impulsivity in at least two areas that are potentially self-damaging; 5. Recurrent suicidal behavior, gestures, or threats, or self-mutilating behavior; 6. Affective instability due to a marked reactivity of mood; 7. Chronic feelings of emptiness; 8. Inappropriate, intense anger or difficulty controlling anger; 9. Transient, stress-related paranoid ideation or severe dissociative symptoms. Factors: F1: Affective Instability and Impulsivity; F2: Interpersonal Unstable and Extreme Reactions; F3: Identity Disturbance. Significant values are indicated in bold.

### BPD traits profile across age groups

**Figure 2:**
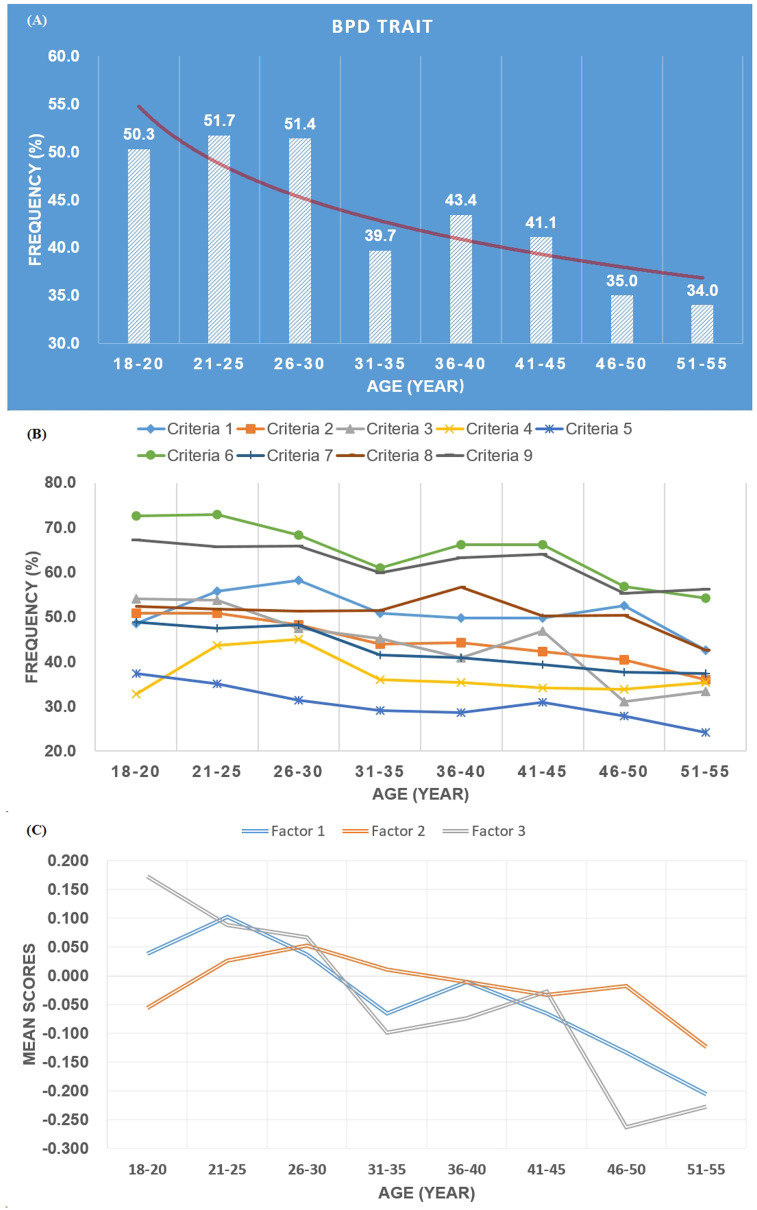
Frequency of BPD trait **(A)**, criteria **(B)**, and profile of three factors **(C)** across age groups.

As shown in **Figure 2**, there is a general decrease in BPD traits, symptoms, and factors with increasing age. Specifically, in **Figure 2A**, the proportion of positive BPD traits is approximately halved before the age of 30 and decreases to around one-third after the age of 30. Likewise, there is a declining trend in the symptoms associated with each diagnostic criterion (**Figure 2B**). Based on the one-way ANOVA indicates statistically significant differences in the declining trends of Factor 1 (*F* = 3.074, *df* = 7, *p*=0.003) and Factor 3 (*F* = 6.413, *df* = 7, *p* < 0.001) with age (**Figure 2C**).

### BPD traits profile across diagnostic groups

Based on the diagnostic grouping of Schizophrenia (N=938), Mood Disorders (N=817), and Anxiety Disorders (N=647), significant differences were observed in the distribution of BPD traits, symptoms, and factors among these three groups (**Table 3**). BPD traits were most common in the Mood Disorders group at 55.7%, followed by the Anxiety Disorders group at 44.4%, and Schizophrenia group at 41.5% (*χ^2^* = 38.084, *p* < 0.001). Among the 9 symptom criteria, except for Criterion 2 where there was no significant difference among the three groups, differences were observed for all other criteria (*p* < 0.05). Specifically, BPD symptoms were most prevalent in the Mood Disorders group, except for Criterion 4 “Impulsivity in at least two areas that are potentially self-damaging,” which was relatively less common in the Anxiety Disorders group. However, Criterion 4 was relatively more prevalent in the Schizophrenia group at 46.1%. Among the three factors, the Mood Disorders group had the highest factor scores, followed by Schizophrenia for factors 1 (*F* = 8.176, *p* < 0.001) and 2 (*F* = 5.986, *p* = 0.003), and Anxiety Disorders for factor 3 (*F* = 14.595, *p* < 0.001).

**Table 3:** Frequency of BPD trait, criteria, and profile of three factors, compared among diagnostic groups.

	Schizophrenia (N=938)	Mood Disorders (N=817)	Anxiety Disorders (N=647)	*χ^2^/F*	*P*
BPD trait (Criteria >=5) [n (%)]	389 (41.5%)	455 (55.7%)	287 (44.4%)	***χ^2^*** = 38.084	**<0.001**
Diagnostic criteria [n (%)]					
Criteria 1	487 (51.9%)	476 (58.3%)	311 (48.1%)	*χ^2^* = 15.839	**<0.001**
Criteria 2	423 (45.1%)	404 (49.4%)	290 (44.8%)	*χ^2^* = 4.332	0.115
Criteria 3	424 (45.2%)	450 (55.1%)	269 (41.6%)	*χ^2^* = 29.898	**<0.001**
Criteria 4	432 (46.1%)	337 (41.2%)	188 (29.1%)	*χ^2^* = 47.181	**<0.001**
Criteria 5	291 (31.0%)	336 (41.1%)	160 (24.7%)	*χ^2^* = 46.182	**<0.001**
Criteria 6	612 (65.2%)	589 (72.1%)	439 (67.9%)	*χ^2^* = 9.527	**0.009**
Criteria 7	373 (39.8%)	434 (53.1%)	283 (43.7%)	*χ^2^* = 32.384	**<0.001**
Criteria 8	431 (45.9%)	461 (56.4%)	348 (53.8%)	*χ^2^* = 20.852	**<0.001**
Criteria 9	576 (61.4%)	550 (67.3%)	430 (66.5%)	*χ^2^* = 7.787	**0.020**
Factors [Mean (SD.)]					
Factor 1	0.022 (1.073)	0.105 (0.974)	-0.108 (0.941)	*F* = 8.176	**<0.001**
Factor 2	-0.015 (1.026)	0.105 (1.040)	-0.072 (0.963)	*F* = 5.986	**0.003**
Factor 3	-0.103 (1.024)	0.150 (0.972)	0.053 (0.967)	*F* = 14.595	**<0.001**

Diagnostic Criteria: 1. Frantic efforts to avoid real or imagined abandonment; 2. A pattern of unstable and intense interpersonal relationships; 3. Identity disturbance; 4. Impulsivity in at least two areas that are potentially self-damaging; 5. Recurrent suicidal behavior, gestures, or threats, or self-mutilating behavior; 6. Affective instability due to a marked reactivity of mood; 7. Chronic feelings of emptiness; 8. Inappropriate, intense anger or difficulty controlling anger; 9. Transient, stress-related paranoid ideation or severe dissociative symptoms. Factors: F1: Affective Instability and Impulsivity; F2: Interpersonal Unstable and Extreme Reactions; F3: Identity Disturbance. Significant values are indicated in bold.

## Discussion

### Key findings

Through a cross-sectional survey involving a substantial clinical sample, this study provides a comprehensive overview of the distribution of BPD pathology among a large clinical population. The findings indicate that approximately half of psychiatric patients exhibit a prominent self-reported trait of BPD, with affective instability being the most common symptom. The clinical features of BPD can be summarized into three dimensions: Affective Instability and Impulsivity, Interpersonal Unstable and Extreme Reactions, and Identity Disturbance. While there was no difference in the proportion of reported BPD traits between genders, suicidal or self-mutilating behavior and extreme reactions were more common among female patients. Regarding age effects, BPD traits, symptoms, and the three dimensions showed an overall declining trend with increasing age. More than half of the patients diagnosed with mood disorders reported BPD traits, and clinical BPD symptoms were more pronounced among them compared to patients with schizophrenia and anxiety disorders. Regarding the impulsivity dimension of BPD symptoms, schizophrenia patients exhibited more pronounced features compared to patients with anxiety disorders.

### Gender effect

The manifestation of BPD features occurs in both genders, yet the debate on whether they are more prevalent in men or women persists in the literature, remaining an empirical question (27). Studies focusing on clinical differences between genders in BPD patients are limited (28). Some studies (29–32) suggest that certain BPD features are more prevalent in women, while others report no significant difference across genders (19, 33), and a minority suggest that some clinical manifestations are more common in men (34, 35). Our study found no difference between genders in self-reported BPD traits. However, female patients exhibited higher prevalence rates compared to male patients in criteria 5 “Recurrent suicidal behavior, gestures, or threats, or self-mutilating behavior”, criteria 8 “Inappropriate, intense anger or difficulty controlling anger”, and criteria 1 “Frantic efforts to avoid real or imagined abandonment”. The differences in societal gender roles may contribute to the observed gender disparities. In many societies, traditional gender norms and expectations shape how individuals express and cope with emotions, interpersonal relationships, and distress. From a young age, boys and girls are often socialized differently in terms of how they are encouraged to express their emotions. Boys may be taught to suppress vulnerable emotions like sadness or fear, while girls may be encouraged to express these emotions more openly. This could lead to differences in how BPD-related emotions such as anger, sadness, or anxiety are expressed and perceived between genders. While our study primarily focused on psychological differences, it is essential to consider the influence of hormonal variations, neurobiological factors, and differences in brain structure and function on the manifestation of BPD. Future research could explore the interplay between psychological and physiological factors to gain a more comprehensive understanding of gender effects in BPD pathology.

### Age effect

Previous studies (36–38) have produced inconclusive evidence regarding whether improvements in BPD symptoms are attributed to the passage of time or differences in the age of individuals in the study. Nonetheless, the potential for age-related reductions in symptoms is supported by research indicating that BPD-relevant symptoms change with age across various psychopathologies (39). For instance, impulsivity and aggression are more strongly associated with completed suicides in younger individuals compared to older ones (40). Our study findings indicate a trend of alleviation in BPD traits, symptoms, and factors with increasing age, especially for the dimension of identity disturbance. This observed trend could be attributed to several factors. As individuals age, they often experience greater maturity and personal growth. This may lead to improved self-awareness, emotional regulation, and coping skills, which are essential components in managing BPD symptoms. With age comes increased stability in various aspects of life, such as relationships, career, and social support networks. Greater stability can provide a buffer against the emotional turmoil and interpersonal difficulties characteristic of BPD, contributing to symptom alleviation (41). Age-related changes in brain structure and function, particularly during adolescence and early adulthood, may contribute to variations in cognitive and emotional processes relevant to BPD pathology. Future research could explore the interplay between neurodevelopmental factors and psychological manifestations to gain a more comprehensive understanding of age effects in BPD.

### Diagnostic effect

BPD exhibits high rates of comorbidity with various Axis I disorders in both clinical (42) and community samples (43, 44). What distinguishes BPD from many other conditions is its shared characteristics with both internalizing and externalizing disorders (45). Regarding internalizing disorders, features of BPD such as affective instability, feelings of emptiness, and interpersonal difficulties may contribute to the elevated rates of comorbidity between BPD and mood (46)/anxiety disorders (47). On the other hand, concerning externalizing disorders, previous research has established a strong association with substance use (48)/impulse control disorders (49). In our current clinical sample, approximately half of the patients reported BPD traits, indicating the widespread presence of BPD symptom dimensions among clinical populations, particularly among patients with mood disorders. This suggests that assessing BPD features and symptoms in clinical patients can provide additional insight into the comprehensive understanding of both internalizing and externalizing disorders. Given the elevated risk of self-harm, suicide, and impulsivity among BPD patients, developing personalized risk management strategies based on the assessment of BPD features may be necessary in psychiatric clinical routine (50).

### Limitations

Several limitations should be considered when interpreting the findings of this study. Firstly, despite being a large-scale study, the cross-sectional design used restricts the ability to establish causal relationships; instead, the study provides descriptive comparisons. Future research could benefit from longitudinal designs, such as cohort studies, to delve deeper into causal connections. Secondly, recall bias is inherent in retrospective assessments, and the accuracy of recollections may be influenced by varying illness trajectories and patient ages. Moreover, axis I psychiatric symptoms may influence the collection of BPD traits and symptoms, potentially leading to fabrication or misconstruction. Although we emphasized during the PDQ-4plus assessment that questions are targeted towards consistent behavioral patterns rather than transient symptoms, patients may still be influenced when self-reporting. For example, individuals with severe depression may be more inclined to perceive themselves as possessing pathological personality traits. Lastly, due to the extensive scope of assessments focusing primarily on BPD traits, the study did not systematically evaluate patients’ clinical symptoms. Consequently, the relationship between different symptom types and BPD traits/symptoms remains unexplored.

## Conclusion

Our study identified widespread presence of BPD traits and symptoms among psychiatric outpatients, with characteristic distributions across gender, age, and diagnostic categories. These findings suggest that clinicians should be vigilant for the presence of BPD traits and symptoms when assessing psychiatric patients, particularly in certain demographic groups such as females, younger individuals and with diagnosis of mood disorders. Early identification and intervention for BPD traits can help prevent the development of more severe symptoms, suicide and self-harm risks and improve overall treatment outcomes.

## Data availability statement

The raw data supporting the conclusions of this article will be made available by the authors, without undue reservation.

## Ethics statement

The studies involving humans were approved by The ethics committee of Shanghai Mental Health Center. The studies were conducted in accordance with the local legislation and institutional requirements. The participants provided their written informed consent to participate in this study.

## Author contributions

YL: Writing – original draft, Resources, Formal analysis, Data curation. ZG: Writing – review & editing, Formal analysis, Data curation, Conceptualization. YZ: Writing – review & editing, Data curation, Funding acquisition. YW: Writing – review & editing, Validation, Investigation. LX: Writing – review & editing, Investigation. XT: Writing – review & editing, Funding acquisition, Formal analysis, Data curation. ZW: Writing – review & editing, Investigation. YH: Writing – review & editing, Formal analysis, Data curation. JW: Writing – review & editing, Visualization, Validation, Supervision, Software, Resources, Conceptualization. HW: Writing – review & editing, Data curation, Resources. YLL: Writing – review & editing. YM: Writing – review & editing, Resources, Methodology, Conceptualization. TZ: Writing – review & editing, Writing – original draft, Validation, Methodology, Investigation, Funding acquisition, Formal analysis, Data curation, Conceptualization.
